# The Role of Dietary Phytochemicals: Evidence from Epidemiological Studies

**DOI:** 10.3390/nu15061371

**Published:** 2023-03-12

**Authors:** Kyong Park

**Affiliations:** Department of Food and Nutrition, Yeungnam University, Gyeongsan 38541, Republic of Korea; kypark@ynu.ac.kr; Tel.: +82-53-810-2879; Fax: +82-53-810-4666

Phytochemicals are biologically active substances derived from plants that play various roles in the human body [1]. To date, over 10,000 types of phytochemicals have been discovered, with their effects on human health depending on the type and structure [2]. The health benefits of phytochemicals have been documented and it is widely recommended to consume plant-based foods rich in these compounds [3,4,5].

Phytochemicals are an important component of the human body, particularly in their role as antioxidants [6,7]. These substances serve as a protective shield for cells, defending them against the harm caused by free radicals [8]. Free radicals are unstable molecules that create oxidative stress, which can lead to cell damage and increase the risk of chronic illnesses such as cancer, cardiovascular disease, and diabetes [9]. The antioxidant properties of certain phytochemicals, such as carotenoids and polyphenols, are especially strong, enabling them to neutralize free radicals and reduce oxidative stress [10].

The results of several meta-analyses demonstrate that higher levels of carotenoids, such as α-carotene, β-carotene, β-cryptoxanthin, lycopene, and lutein/zeaxanthin, and polyphenols in the diet or plasma are linked to lower frailty [11] and a reduced risk of cardiovascular disease [12,13,14]. In addition, terpenes, present in nuts and crude pressed oils, have been associated with improved cardiovascular health [15], and phytosterols, which can be found in nuts and unrefined pressed oils, have been linked to cholesterol reduction [16].

Although some progress has been made, the research has mainly focused on a limited number and types of phytochemicals in epidemiological studies. Moreover, studies examining the effect of dietary phytochemical intake on human health through community-based cohort studies have been particularly scarce. This lack of information is due to the scarcity of food composition databases, which provide crucial details about these metabolites needed to test hypotheses about the health benefits of specific plant metabolites in human population. Generally, plant-based foods that are rich in phytochemicals include whole grains, vegetables and fruits, nuts, and legumes. As a result, many epidemiological studies have evaluated the effects of single plant-based foods, food groups, or dietary patterns that are rich in phytochemicals. 

Recently, a new dietary index, phytochemical index (PI), has been developed to efficiently evaluate the health effects of phytochemical-rich foods in large population-based epidemiological studies. PI serves as a convenient surrogate measure of phytochemical intake. A growing number of studies have investigated the effects of the PI on chronic diseases such as obesity [17], low-grade inflammation [18], hypertension [17], metabolic syndrome [19,20], diabetes [21], and breast cancer [22,23,24]. Despite the benefits of using the PI tool to assess phytochemical intake levels in population-based studies, it is important to recognize the limitations of such research. The fact that most of these studies have been conducted in Iran and to a lesser extent in Korea means that their findings may not be generalizable to other populations and regions. Additionally, the PI calculation method based on food source calorie may lead to underrepresentation or omission of low-calorie, phytochemical-rich foods such as green tea, even though they are significant sources of these compounds and have known health benefits. Given these limitations, it is imperative to conduct further research to enhance our comprehension of the impact of phytochemicals on chronic diseases. One way to achieve this is through the development of a new index that can account for the limitations of the PI and provide a more comprehensive measure of phytochemical intake. By conducting studies using this new index across diverse populations and geographical locations, we can gain a deeper understanding of the impact of phytochemical-rich foods on human health and develop more effective dietary recommendations that are applicable to a wider range of populations and regions. Through such research, we can identify the optimal intake levels of phytochemicals for different populations and help people make informed choices about their diet to improve their overall health and well-being.

In conclusion, the significance of consuming phytochemical-rich foods for human health cannot be overstated. The development of the PI has simplified the assessment of phytochemical intake levels in population-based studies, thereby aiding research into the connection between phytochemicals and human health. However, it is clear that further studies are necessary to obtain a deeper understanding of the health benefits of individual plant metabolites and the overall impact of phytochemical intake. This calls for larger and more diverse population-based studies that account for the cultural dietary practices of different groups of people. Such research will enable the identification of the role of phytochemicals in promoting health in various geographic regions, ultimately facilitating the development of effective dietary interventions and recommendations.
